# Preliminary Characterization of NP339, a Novel Polyarginine Peptide with Broad Antifungal Activity

**DOI:** 10.1128/AAC.02345-20

**Published:** 2021-07-16

**Authors:** Vanessa Duncan, Daniel Smith, Laura Simpson, Emma Lovie, Laura Katvars, Leon Berge, Jennifer Robertson, Shane Smith, Carol Munro, Derry Mercer, Deborah O’Neil

**Affiliations:** a NovaBiotics, Ltd., Aberdeen, United Kingdom; b The University of Aberdeen, Institute of Medical Sciences, Aberdeen, United Kingdom

**Keywords:** antifungal agents, antifungal resistance, antifungal therapy, antimicrobial peptides

## Abstract

Fungi cause disease in nearly one billion individuals worldwide. Only three classes of antifungal agents are currently available in mainstream clinical use. Emerging and drug-resistant fungi, toxicity, and drug-drug interactions compromise their efficacy and applicability. Consequently, new and improved antifungal therapies are urgently needed. In response to that need, we have developed NP339, a 2-kDa polyarginine peptide that is active against pathogenic fungi from the genera *Candida*, Aspergillus, and Cryptococcus, as well as others. NP339 was designed based on endogenous cationic human defense peptides, which are constituents of the cornerstone of immune defense against pathogenic microbes. NP339 specifically targets the fungal cell membrane through a charge-charge-initiated membrane interaction and therefore possesses a differentiated safety and toxicity profile to existing antifungal classes. NP339 is rapidly fungicidal and does not elicit resistance in target fungi upon extensive passaging *in vitro*. Preliminary analyses in murine models indicate scope for therapeutic application of NP339 against a range of systemic and mucocutaneous fungal infections. Collectively, these data indicate that NP339 can be developed into a highly differentiated, first-in-class antifungal candidate for poorly served invasive and other serious fungal diseases.

## INTRODUCTION

Fungal diseases remain a significant area of unmet medical need globally and affect close to one billion individuals worldwide. They range from mucosal infections to serious life-threatening invasive fungal diseases (IFDs) and are mainly caused by fungi from the genera *Candida*, Aspergillus, Cryptococcus, Pneumocystis, and *Histoplasma* and mucormycetes (*Mucorales*). Staggeringly, mortality associated with fungal diseases is comparable with that of tuberculosis and three times higher than that of malaria (1). While superficial fungal infections can occur in relatively healthy individuals, IFDs predominantly affect immunosuppressed and critically ill individuals (e.g., solid organ transplant recipients, cancer patients, and HIV patients). Paradoxically, some major advances of modern medicine and some invasive procedures come at a cost of increased IFD incidence (2).

Despite a major impact on human health, fungal diseases have failed to garner as much attention from the policymakers and industry as other infectious diseases. For instance, compared with over 15 classes of antibacterial drugs (3), only five classes of antifungal agents are currently available, with only three of them in mainstream clinical use (for reviews, see, e.g., references 4 and 5). Furthermore, the use of current antifungals is often limited by adverse side effects (e.g., amphotericin B is nephrotoxic, and azoles are hepatotoxic) (6, 7) and drug-drug interactions (e.g., interactions of statins and fluconazole and interactions of posaconazole with some immunosuppressants) (8). Furthermore, these drugs can sometimes be fungistatic instead of fungicidal, which drives the development of antifungal resistance (9).

Antifungal drug resistance is a major and multifactorial problem. First, in addition to the fungistatic effect, the lack of tools for accurate and timely diagnosis of infection necessitates prophylactic use and empirical therapy, risking prolonged exposure of the pathogen to suboptimal concentrations of the drug, driving resistance development (5, 10–12). Furthermore, since the available antifungals typically target a specific enzyme or cellular pathway, the pathogen can become resistant by modifying or overexpressing the drug target or by inducing the production of efflux pumps that remove the drug from the cell (13). Cross-resistance to antifungal drugs has been observed (14). In addition, agricultural and industrial fungicides that have a similar structure and/or mechanism of action to those of drugs used in the clinic can also drive the evolution of resistance (15–18). These issues not only limit the efficacy of drugs against existing pathogens, but also shift the epidemiology toward the emergence of new pathogens that are inherently resistant to currently available antifungal agents (19–23). New antifungal molecules are therefore urgently needed to combat drug resistance and emerging fungal pathogens.

To date, the majority of antifungal drug efforts have generally focused on the improvement of existing molecule classes: e.g., reducing their toxicity, improving pharmacodynamics and pharmacokinetics, enhancing their formulations, and increasing specificity (4, 24). In contrast, we are focused on harnessing nature’s own defenses to fight microbial infections. Specifically, we have been evaluating the antifungal potential of synthetic peptides inspired by endogenous host defense peptides (HDPs), which are short (50 to 100 amino acids) cationic and amphipathic peptides that play a critical “first responder” role in immune defense and have broad-spectrum antimicrobial activity (25–28).

Here, we present preliminary characterization of NP339, a 2-kDa polyarginine peptide for the treatment of IFDs and other serious fungal infections. We show that NP339 has a broad spectrum of antifungal activity in artificial medium and biological matrices, does not elicit resistance development *in vitro*, has an excellent *in vitro* safety and toxicity profile, and is well tolerated and shows initial efficacy in rodent models of fungal diseases. NP339 is a promising candidate molecule representing a new class of therapy for poorly served fungal infections.

## RESULTS

### Discovery of NP339, a polyarginine peptide with antifungal activity.

HDPs are naturally occurring defense peptides and a cornerstone of immunity. They act by eliciting direct antimicrobial effects against microbes and/or immunopotentiation (27, 28). We aimed to exploit only the HDP membrane-disrupting potential as a mechanism of antifungal activity for a novel antimicrobial peptide. HDPs are highly evolutionarily conserved and contain a high percentage (30 to 50%) of positively charged residues, such as arginines and lysines. The peptides align, via these residues, with the negatively charged microbial membrane and perturb it (27, 29). We asked what would happen if such a peptide would be stripped to contain only cationic residues (e.g., arginine and lysine). Short, synthetic polyarginine peptides have already been developed as cell-penetrating peptides to deliver various cargoes (drugs or dyes) to the mammalian cell (30, 31), but to the best of our knowledge, their antifungal potential had not yet been explored. We exposed Candida albicans to various cationic polypeptides generated by solid-phase synthesis as acetate salts (Table 1). While polylysines and short peptides (Arg9 and shorter) did not kill the target cell, longer arginine chains within the range expected from the cationic residue composition of endogenous HDPs, and optimally with a molecular mass of approximately 2 kDa, had pronounced antifungal activity. This was a novel observation, and we therefore proceeded to investigate the antifungal activity of polyarginine peptides in more detail. We selected a 2-kDa polyarginine peptide (henceforth referred to as NP339) for further, in-depth analysis.

**TABLE 1:** Antifungal activity of polyarginine peptides

Sequence	Length (aa)	MIC (μM)^*a*^
RRR	3	>2,048
RRRRR	5	>2,048
RRRRRRR	7	>2,048
RRRRRRRRR	9	2,048
RRRRRRRRRRR	11	8
RRRRRRRRRRRRR^*b*^	13	2
RRRRRRRRRRRRRRR	15	2

a MIC values against C. albicans NCTC 3179 are shown. The data are representative of one replicate.

b NP339.

### NP339 specifically targets the fungal cell membrane in a charge-dependent manner.

Cationic antimicrobial peptides interact with and disturb the microbial membrane, while cell-penetrating peptides interact with the membrane without destroying it. We therefore first confirmed the mode of NP339 antifungal activity.

**FIG 1:**
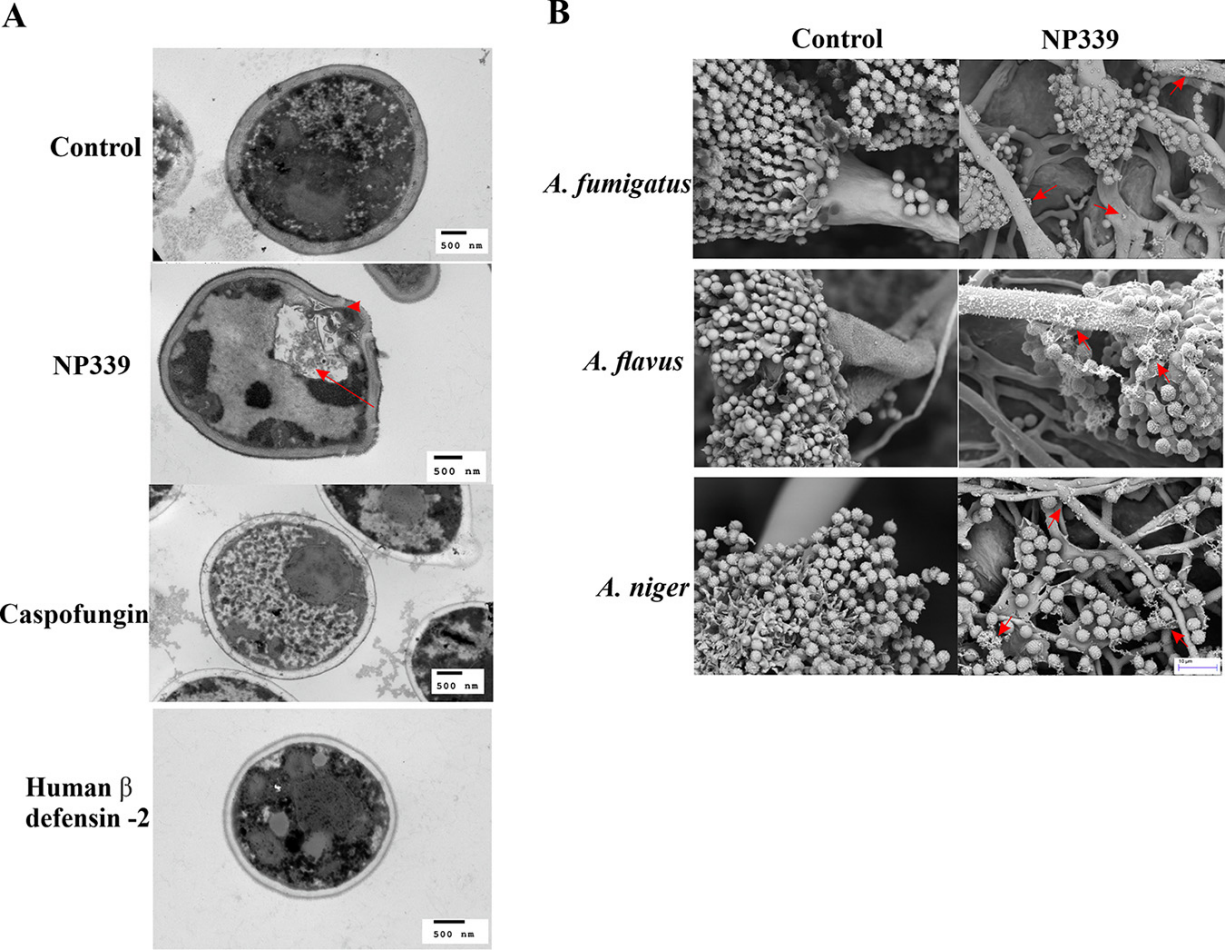
NP339 destroys planktonic and biofilm-forming fungal cells. (A) C. albicans AM2003-0182 cells were grown in 2× RPMI 1640 medium at 30°C for 1 h. The cells (1 × 10^8^ CFU/ml) were exposed to the indicated agents for 30 min and analyzed by TEM. The doses tested were 4 mg/liter NP339, 0.5 mg/liter caspofungin, and 0.25 mg/liter human β-defensin 2. The images are representative of at least five fields of view. An arrow points to loss of cellular contents. An arrowhead points to the site of cell membrane and cell wall rupture. (B) The indicated Aspergillus strains were grown in RPMI 1640 medium on a 0.2-μm-pore Whatman polycarbonate filter for 48 h at 35°C.The cells were exposed to 250 mg/liter NP339 for 1 h and analyzed by SEM. The images are representative of at least two fields of view. Arrows point to released cellular content. No released cellular content is visible in untreated (control) samples.

Electron microscopy analysis indicated that, similar to one of the known functions of endogenous HDPs, NP339 acts by causing cell lysis. As observed by transmission electron microscopy (TEM), NP339 exposure resulted in the lysis of planktonic cells of C. albicans (Fig. 1A). Specifically, exposure to 4 mg/liter (1× MIC of NP339 against this strain of C. albicans determined by standard methods of the Clinical and Laboratory Standards Institute [CLSI]) (32) of NP339 resulted in rapid cell destruction within 30 min: rupture of the cell membrane and wall, with the resulting loss of cellular contents. This effect was not observed upon exposure to even 1× MIC of caspofungin or human β-defensin 2, or double-distilled water (the control) for the same time period. Furthermore, NP339 (33) appeared to also perturb the surface of and/or lyse biofilm-forming Aspergillus cells, as indicated by cellular debris formed in the presence of NP339, observed by scanning electron microscopy (SEM) (Fig. 1B).

**FIG 2:**
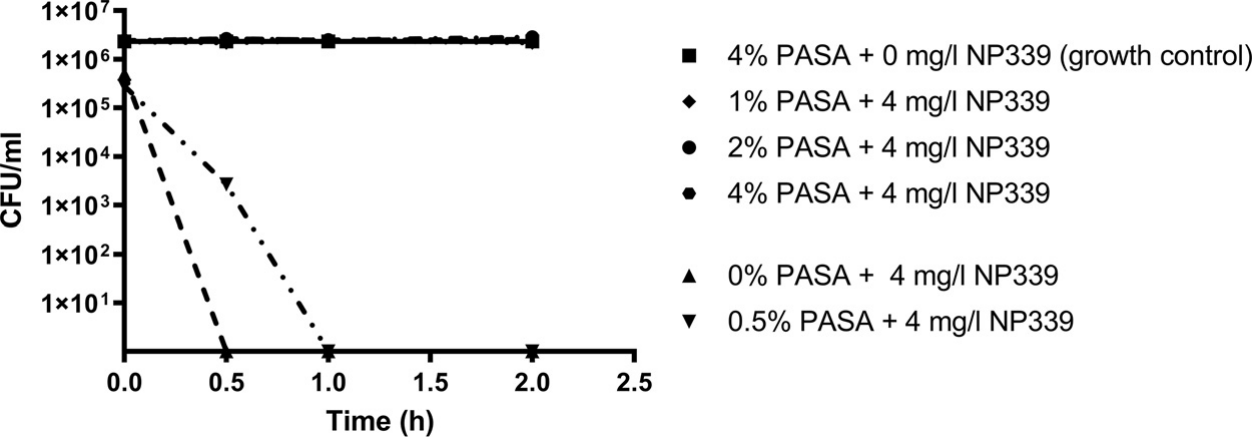
Antifungal activity of NP339 is charge dependent. NP339 was incubated with different concentrations of PASA for 30 min at 37°C in sdH_2_O. C. albicans SC5314 cells were added to pretreated NP339 and incubated for the indicated periods of time at 37°C. Afterwards, the cells were plated and the resultant colonies counted. The data are presented as means ± standard error (SE) (*n* = 3).

Furthermore, to confirm the charge dependency of NP339 activity, we incubated NP339 (at a concentration equivalent to the MIC as determined by standard CLSI methods) with polyanetholesulfonic acid (PASA), a negatively charged molecule that binds to and effectively neutralizes the functional cationic charge of cationic compounds. We then evaluated the effectiveness of PASA-preincubated peptide against C. albicans (Fig. 2). As anticipated, preincubation with PASA dramatically reduced the fungicidal activity of NP339. Specifically, while the MIC (4 mg/liter) of NP339 killed C. albicans within 30 min of exposure, that time was extended to 60 min for NP339 pretreated with 0.5% PASA. Under saturated conditions (incubation of NP339 with >1% PASA), NP339 activity was completely inhibited. These observations confirmed the criticality of electrostatically mediated membrane interactions for NP339 activity. Of note, PASA anionicity exceeds that of physiological salt concentrations, whereas the tissue culture medium in which MIC assays were performed and in which NP339 is active is equivalent to physiological salt conditions. We also show data (below) that NP339 retains activity in human whole blood and saliva. Activity in the biological matrices and under physiological salt conditions is a key element of NP339 that supports its viability as a potential drug candidate, whereas a number of antimicrobial peptides investigated by other groups have not had these essential properties (34, 35).

**FIG 3:**
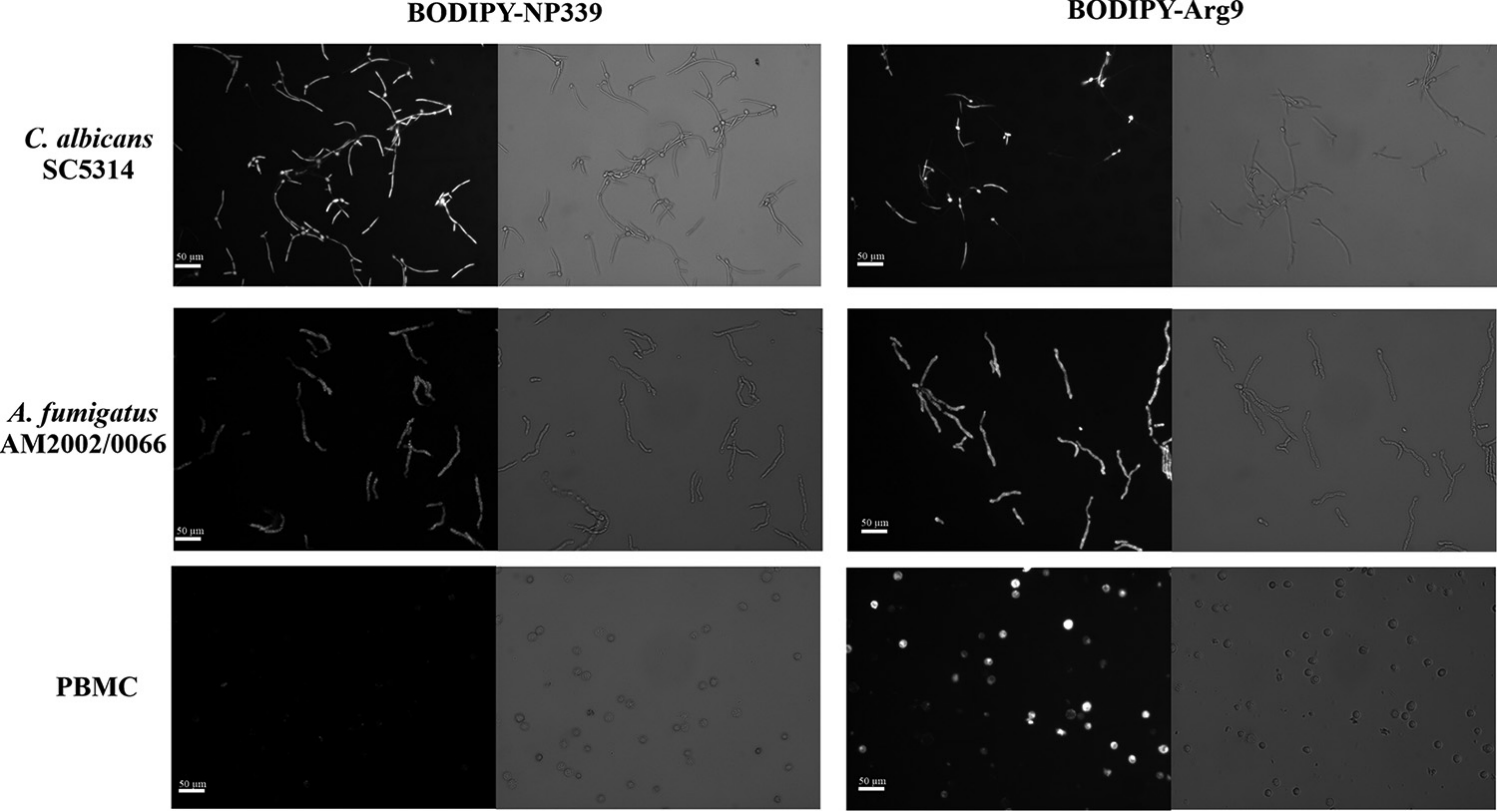
NP339 specifically targets the fungal cell. C. albicans SC5314 and A. fumigatus AM2002/0066 cells were prepared in RPMI 1640 medium, as described in Materials and Methods. PBMCs were isolated from fresh human whole blood. The cells (1 × 10^5^ CFU/ml) were mixed with 20 μM BODIPY-labeled peptides and incubated at 37°C for 1 h. The samples were then analyzed by optical and fluorescence microscopy. The images are representative of three independent replicates.

Since the interaction of NP339 with the fungal cell membrane is charge dependent, we anticipated that the peptide would not interact, or at least interact differently, with the mammalian cell membrane. That is because the lipid compositions, which affect membrane charge and interactions with antimicrobial peptides (29, 36), of the fungal and mammalian cell membranes are different. Accordingly, we labeled the C terminus of NP339 with the fluorophore 4,4-difluoro-5,7-dimethyl-4-bora-3a,4a-diaza-*s*-indacene-3-propionyl ethylenediamine (BODIPY FL EDA). BODIPY labeling did not affect the antifungal activity of the peptide (data not shown). We then used fluorescence microscopy to evaluate the interaction between BODIPY-labeled NP339 and the fungal and human peripheral blood mononuclear cells (PBMCs). As a control, we also BODIPY labeled and tested the cell-penetrating peptide Arg9, which enters the mammalian cell without destroying it (31). Indeed, while the BODIPY-labeled Arg9 peptide penetrated both cell types tested, BODIPY-labeled NP339 only penetrated the fungal cell (Fig. 3). These observations confirmed the specificity of NP339 for the fungal cell with no discernible host cell interaction.

### NP339 has a broad antifungal activity, is rapidly fungicidal *in vitro*, and is active in various biological matrices.

We next investigated the spectrum of NP339 activity and compared its performance *in vitro* with that of standard-of-care antifungals. Extensive screening revealed a broad spectrum of NP339 activity (see Table S1 in the supplemental material). The peptide was active against fungal pathogens from such genera as *Candida*, Aspergillus, Cryptococcus, and *Exophiala*, as well as others. As a caveat, the actual minimally active concentrations of NP339 might be much lower than those determined using the standard CLSI and EUCAST antimicrobial susceptibility test methods, which are designed for compounds with discrete intracellular targets and therefore underestimate, or do not reflect at all, the apparent efficacy of membrane-targeting antimicrobial agents such as antimicrobial peptides (AMPs) (37).

Furthermore, time-kill experiments with representative fungal pathogens revealed that NP339 acted much more rapidly than the other antifungals tested (Table 2). For example, NP339 concentration equivalent to 2× MIC (by CLSI methods) resulted in 3-log reduction of C. albicans cell numbers after less than 1 h of exposure. In comparison, it took 4 h for a caspofungin concentration equivalent to 4× MIC (by CLSI methods) to achieve that effect. Of note, NP339 was as effective as amphotericin B and more effective than fluconazole and caspofungin against the emerging fungal pathogen Candida auris. Furthermore, while NP339 did not kill Aspergillus fumigatus conidia as rapidly as it did *Candida* cells, the killing was nonetheless more rapid than that elicited by the other agents tested (Table 2). Overall, NP339 killed various fungal cells much more rapidly than existing antifungal agents.

**TABLE 2:** Comparisons of time-kill results for NP339 and standard-of-care antifungals against representative fungal pathogens

Fungus	Antifungal	Final concn		Time of kill (h)^*a*^
		mg/liter	×MIC	
C. albicans (*n* = 2)	NP339	4	2	0.5
	Fluconazole	32	32	6
	Caspofungin	1	4	4

C. auris (*n* = 1)	NP339	32	16	4
	Amphotericin B	32	16	4
	Fluconazole	256	16	48<
	Caspofungin	16	16	6–24

A. fumigatus				
Conidia (*n* = 2)	NP339	256	16	2–4
	Amphotericin B	2–8	4–16	48
	Voriconazole	2–8	16	48
	Posaconazole	4	16	24
	Caspofungin	2–32	1–16	48
8-h germlings (*n* = 2)	NP339	32	2	2–4

a Time taken to kill 3 logs of *Candida* spp. (starting inoculum size 1 × 10^6^ to 5 × 10^6^ cells/ml), or 2 logs of A. fumigatus conidia (starting inoculum size 1 × 10^5^ cells/ml) and the same inoculum germinated for 8 h.

**FIG 4:**
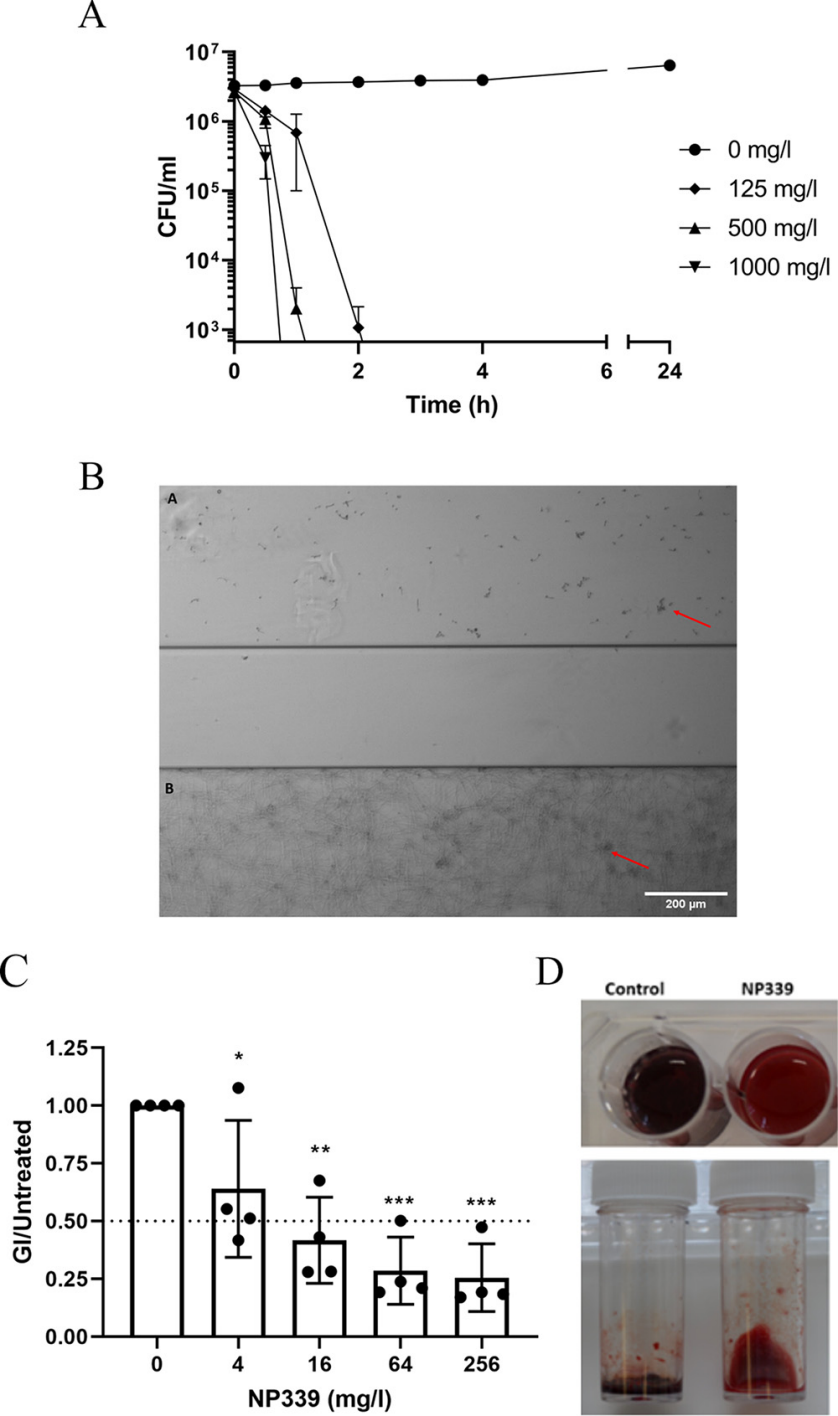
NP339 is active in the human saliva and blood. (A) C. albicans MD cells were incubated in 90% human saliva (final cell density of a 0.5 McFarland standard) and the indicated concentrations of NP339 at 30°C. Samples were withdrawn at the specified time periods, plated on SAB agar, and incubated at 30°C. The resultant cell growth (CFU/ml) is shown. The data are representative of the mean ± SE from triplicate experiments. (B) C. albicans MD cells were exposed to flowing 90% human saliva, in the presence or absence of NP339 (125 mg/liter), in a Fluxion BioFlux 200 (Fluxion, South San Francisco, CA, USA) for 20 h at 37°C. Arrows indicate fungal cell clusters. Scale bar, 200 μm. The images are representative of triplicate experiments. (C) Citrated human whole blood was exposed to A. fumigatus AM2002/0066 conidia (final density, 1 × 10^5^ conidia/ml) for 24 h at 37°C, with or without NP339. The GI was then determined in the sample supernatant (diluted 1:100 in sdH_2_O) by ELISA. The ratio of GI in NP339-treated samples to that in NP339-untreated samples is shown. The data are presented as means ± standard deviation (*n* = 4) (*, *P* < 0.05, **, *P* < 0.01, and ***, *P* < 0.001, versus no-NP339 control by one-way ANOVA with Dunnett’s multiple-comparison test). Dotted line, 50% reduction in GI relative to sham-treated control. (D) Images of citrated human whole blood incubated with A. fumigatus AM2002/0066 conidia, prepared as in panel C, after 48 h of incubation at 37°C, with or without 250 mg/liter NP339 (top) or 500 mg/liter NP339 (bottom). The images represent multiple, consistent observations.

We next asked about the activity of NP339 in biological matrices. Although the growth media used for the time-kill determinations described above largely mimic physiological salt concentrations, they do not reproduce the complexity of the infection site. Biological fluids also contain multiple charged compounds, such as amino acids, proteins, and other substances (37) that could interact with and/or quench the NP339 charge, affecting its activity. Accordingly, we investigated the activity of NP339 in human saliva and whole blood. First, we incubated C. albicans MD cells with 90% saliva in the presence of different concentrations of NP339. Under these conditions, C. albicans MD cells were rapidly killed by supra-MICs of NP339, with a 3-log reduction in CFU/ml within 1 h (for 1,000 mg/liter NP339) or 2 h (for 125 and 500 mg/liter NP339) (Fig. 4A). Furthermore, 125 mg/liter NP339 prevented the attachment and biofilm formation by C. albicans MD cells in flowing saliva experiments that mimic the conditions in the oral cavity (Fig. 4B). These observations confirmed that NP339 is active in the saliva.

To test NP339 activity in the blood, we incubated A. fumigatus AM2002/0066 conidia with human whole blood in the presence of different concentrations of NP339 and quantified galactomannan antigen content in cultures as a readout of efficacy. NP339 exposure significantly reduced the galactomannan index (GI) in samples inoculated with A. fumigatus, indicating that NP339 retains activity in blood (Fig. 4C). In addition, while the blood became dark and clotted in the infected samples in the absence of NP339, no such changes were apparent in the presence of NP339 (Fig. 4D). This further confirmed successful killing of A. fumigatus by NP339 in whole blood. To our knowledge, this is the first example of the antimicrobial activity of AMPs intended for systemic administration being demonstrated *ex vivo* in “whole” human blood (dilution factor from inoculum and peptide solution meaning 85% whole blood). Indeed, activity was not retained but “improved” compared to MIC data obtained from culture media. Importantly, the effective NP339 concentrations are not cytotoxic or hemolytic (see below).

Collectively, the above observations indicate that NP339 has a broad antifungal activity and is rapidly fungicidal *in vitro*. Furthermore, in addition to showing that NP339 is active under physiological salt conditions (see above), we showed that NP339 is also active in complex biological matrices.

### NP339 does not elicit development of cross-resistance *in vitro*.

Exposure to antifungal drugs drives fungal resistance development (14, 38). We therefore next asked whether prolonged exposure to NP339 would induce anti-NP339 resistance in fungi. We anticipated that that would be unlikely, considering the fungal membrane targeting and fungicidal mode of action of NP339.Accordingly, we passaged representative strains of C. auris, C. albicans, Candida glabrata, and Candida tropicalis in media containing sub-MICs (0.25× MIC) of NP339, caspofungin, fluconazole, or amphotericin B (Table 3). The MIC values were determined every 5 passages, up to passage 30, and fold changes from the starting value were calculated. The passaging induced fluconazole resistance in all strains tested, while the effect of caspofungin and amphotericin B on the development of resistance was strain dependent. However, the passaging did not induce NP339 resistance in any strain tested. In fact, the NP339 MIC against C. glabrata AM2007/0125 decreased with passaging.

**TABLE 3:** Effect of extensive passaging of *Candida* species in the presence of sub-MICs of various antifungal compounds on the MICs of these compounds

Strain	Fold change in antifungal MIC at passage 30^*a*^			
	NP339	Caspofungin	Fluconazole	Amphotericin B
C. albicans SC5314	1	4	256	4
C. auris DSMZ1092	1	1	4	4
C. glabrata AM2007/0125	0.125	1	2	1
C. tropicalis AM2005/0568	1	8	256	4

a The MIC of each compound tested at passage 0 was normalized to 1, and the fold change after 30 passages in 0.25× MIC of each antifungal was calculated. A value of 1 signifies no change. The data are averages from three independent replicates.

In addition, where resistance developed for the standard-of-care therapies, we subsequently tested the susceptibility of these strains to NP339. All strains remained susceptible to NP339, indicating that NP339 was effective even against strains with newly acquired resistance to other antifungal agents (Table 4).

**TABLE 4:** Effect of extensive passaging of *Candida* species in the presence of sub-MICs of various antifungal compounds on the NP339 MIC

Strain	Fold change in NP339 MIC at passage 30^*a*^			
	NP339	Caspofungin	Fluconazole	Amphotericin B
C. albicans SC5314	1	1	1	1
C. auris DSMZ1092	1	1	1	1
C. glabrata AM2007/0125	0.031	0.062	1	1
C. tropicalis AM2005/0568	1	1	1	1

a The MIC of NP339 at passage 0 was normalized to 1, and the fold change in the MIC of NP339 after 30 passages in 0.25× MIC of the indicated antifungal was calculated. A value of 1 signifies no change. The data are averages from three independent replicates.

Collectively, these observations indicated that NP339 does not elicit resistance or cross-resistance to standard-of-care antifungals *in vitro*.

### NP339 is not cytotoxic or hemolytic *in vitro*.

**FIG 5:**
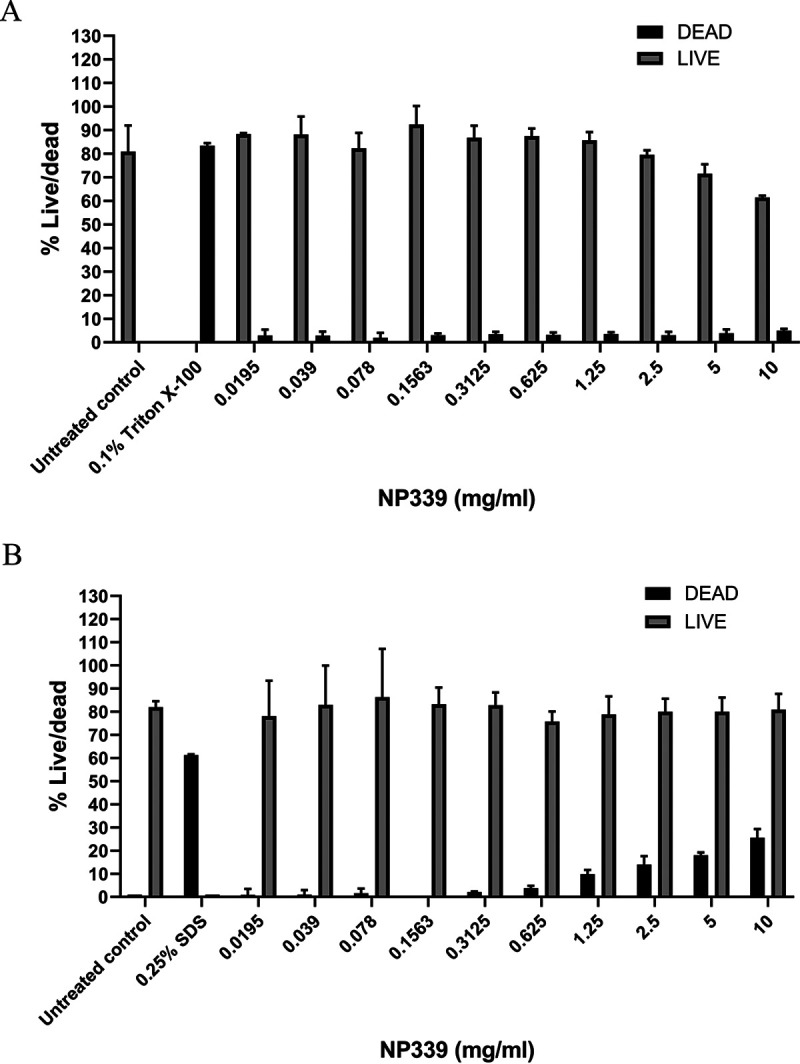
NP339 is not cytotoxic. PBMCs (5 × 10^5^ cells/ml) isolated from the human whole blood (A) and A545 lung epithelial cells (monolayer) (B) were incubated with the indicated concentrations of NP339 for 2 or 3 h, respectively, in RPMI 1640 medium at 37°C. The numbers of surviving cells were determined by LIVE/DEAD staining. The data are shown as means + SD (*n* = 2). The kill controls were 0.1% (vol/vol) Triton X-100 in panel A and 0.25% (wt/vol) SDS in panel B.

Having established the unique fungal cell specificity of NP339, we next investigated the possible cytotoxic effects of NP339 on mammalian cells that would have been exposed to NP339 upon systemic administration (the main mode of antifungal drug administration to individuals with IFD) (39). We first exposed human PBMCs and the A549 epithelial lung cell line to concentrations of NP399 that were hundreds of folds higher than those that elicit antifungal effects *in vitro* (Fig. 5A and B) and determined the live/dead cell ratio using a commercial kit (see Materials and Methods). NP339 was not toxic to these cells even at very high concentrations beyond any proposed therapeutic range.

We then evaluated the hemolytic potential of NP339. The percentage of hemolysis of human red blood cells was negligible: approximately 1% hemolysis upon exposure to up to 0.3 mg/ml NP339 and approximately 2% hemolysis upon exposure to 0.6 to 5 mg/ml NP339 (data not shown). In comparison, incubation with the detergent-positive control resulted in 100% hemolysis (data not shown).

Collectively, the above observations indicated a unique safety and toxicity profile of NP339 as anticipated from a molecule of endogenous provenance.

### Therapeutic efficacy potential of NP339 *in vivo*.

**FIG 6:**
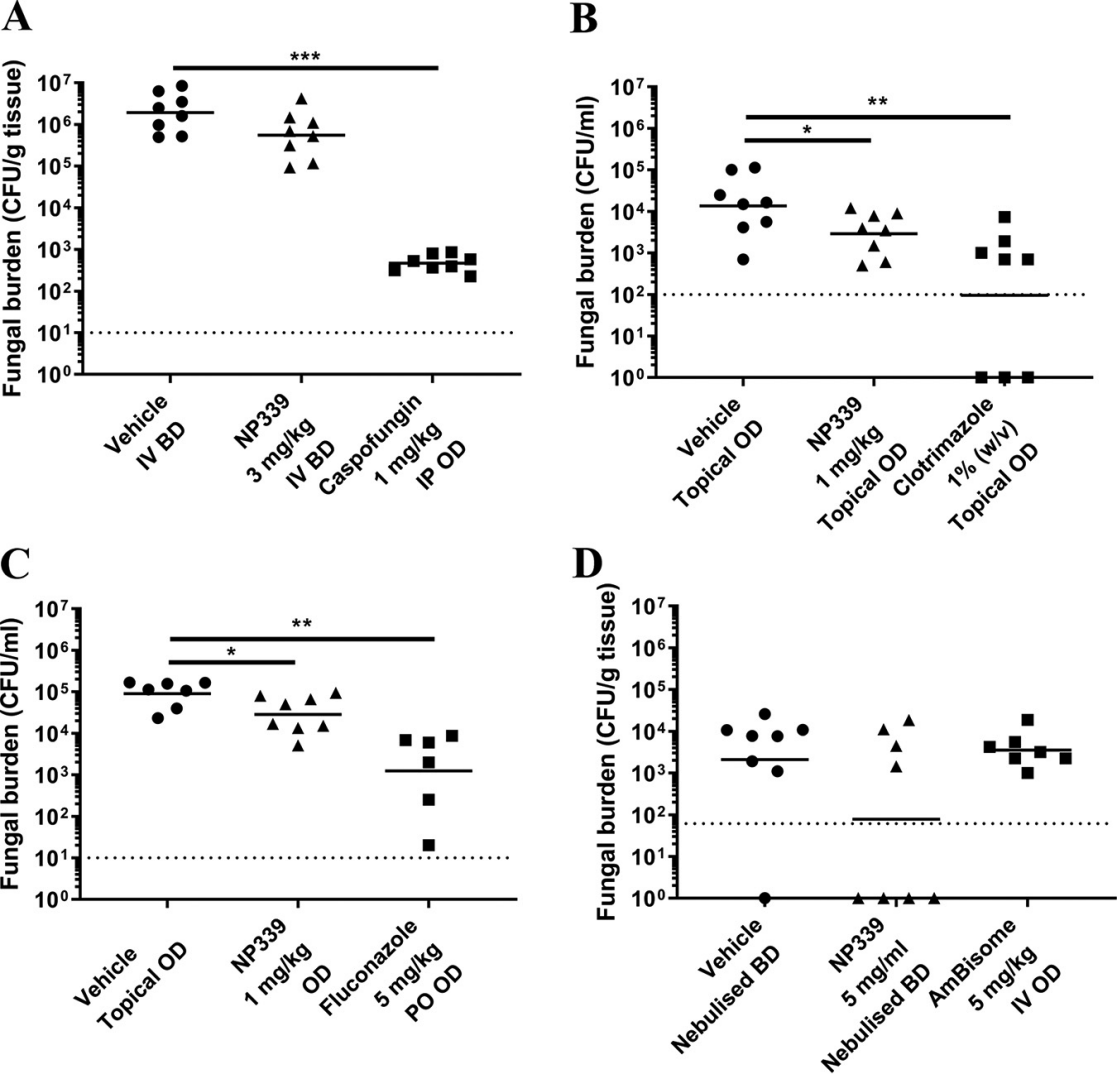
Therapeutic effect of NP339 in murine models of fungal diseases. (A) Murine model of disseminated candidiasis. CD1 male mice (*n* = 8/group) were infected with C. albicans FA6862 (approximately 1.1 × 10^4^ CFU/mouse) by intravenous injection into the lateral tail vein. The indicated agents were administered as specified. Fungal kidney burdens were assessed 29 h postinfection. (B) Murine model of vaginal candidiasis. Female BALB/c mice (*n* = 8/group) were infected with C. albicans 529L (4.5 × 10^6^ CFU/mouse) topically by intravaginal injection under temporary inhaled anesthesia. The indicated agents were administered intravaginally. Fungal burdens in vaginal lavage samples were determined after 3 days. (C) Murine model of oropharyngeal candidiasis. Male CD1 mice (*n* = 8/group) were topically infected with C. albicans FA6862 (approximately 4.0 × 10^8^ CFU/ml) under temporary inhaled anesthesia. The indicated agents were then administered topically, unless otherwise stated. Fungal burdens in the oral swab fluid were determined after 4 days. (D) Murine model of invasive pulmonary aspergillosis. Immunosuppressed male CD1 mice (*n* = 8/group) were infected by intranasal administration of A. fumigatus A1163 (7.2 × 10^4^ CFU/mouse) under temporary inhaled anesthesia. The indicated agents were then administered as specified. Fungal lung burdens were determined at the clinical endpoint. In all panels, individual data points are presented with the geometric mean (*, *P* < 0.05, and **, *P* < 0.01, by Mann-Whitney test). Administration mode: IP, intraperitoneal injection; IV, intravenous injection; PO, oral administration. Administration frequency: OD, once daily; BD, twice daily. Dotted horizontal lines identify the lower limit of detection in each assay.

Finally, we tested the therapeutic potential of NP339 *in vivo* in murine models of fungal disease (Fig. 6). While we observed a trend, but not a significant effect of NP339 administration on the fungal kidney burden in the disseminated candidiasis model (Fig. 6A), there was a significant improvement in the vaginal lavage burden (Fig. 6B) in the vaginal candidiasis model and in the oral swab fluid burden in the oropharyngeal candidiasis model (Fig. 6C). Similarly, nebulized NP339 reduced fungal lung burden in an invasive pulmonary aspergillosis rodent model (Fig. 6D). Of note, the above evaluations were based on fungal tissue burden determination by plating of homogenized tissue. As a caveat, this method is best suited for assessing the efficacy of drugs internalized and retained by the cell, not membrane-acting agents such as NP339. The peptide-fungal cell interaction will be lost during tissue preparation for plating, resulting in an overestimation of the actual fungal burden and hence, underestimation of peptide activity.

Overall, these preliminary observations from models not in any way optimized for peptide therapeutics (mode of delivery, particularly for systemic administration, and fungal burden analysis from tissue as endpoints) were encouraging and suggest the therapeutic potential of NP339 *in vivo*. Further data will be generated in optimized (i.e., determination of burden with appropriate methods) *in vivo* models of infection, in which antimicrobial peptide drugs such as NP339 can be tested more robustly (e.g., with the mode and frequency of systemic administration more representative of clinical use, etc.).

## DISCUSSION

We present our preliminary characterization of NP339, a synthetic 2-kDa polyarginine peptide with broad antifungal activity. NP339 specifically targets the fungal cell membrane, is rapidly fungicidal and active in various biological matrices, does not induce resistance development *in vitro*, and shows initial evidence of efficacy in rodent models of fungal disease. Therefore, it is a promising new candidate molecule for targeting IFDs and other fungal infections.

The structure and mechanism of action of NP339 are unlike those of any existing antifungal drug, as its design is inspired by HDP structure and function. Similar to HDPs (27–29, 40, 41), the activity of NP339 is charge dependent, and the peptide only targets the fungal cell membrane. Furthermore, the observation that NP339 did not penetrate the mammalian cell is in agreement with the known properties of oligoarginines. Futaki et al. (31) determined an apparent size cutoff for oligoarginine peptides, above which the efficiency of penetrating the mammalian membrane decreases (9 amino acids). Accordingly, NP339 is >30% larger than the optimal translocating peptide. Importantly, penetration does not equate to disruption, as the short polyarginine peptide Arg9 penetrated both fungal and mammalian cells tested, yet did not destroy these cells.

Development of fungus-specific drugs is challenging given the similarity between the fungal and mammalian cells. Indeed, off-target effects often result in drug toxicity (4, 24). NP339 specificity for the fungal cell is most likely associated with the different lipid compositions of the fungal and mammalian cell membranes: while mammalian membranes contain cholesterol, fungal membranes do not; the major sterol in the fungal cell membrane is ergosterol (42, 43). Both cholesterol and ergosterol affect membrane fluidity and permeability, as well as interactions with antimicrobial peptides (29, 36, 44, 45).

Considering the specific mode of NP339 activity (i.e., charge-based interactions with the fungal cell membrane), it was not surprising that this peptide was not cytotoxic *in vitro*. Further, NP339 did not elicit a nonspecific immune response, as determined by cytokine measurements in human whole blood exposed to the peptide (data not shown). Indeed, we do not expect this polyarginine peptide to be immunogenic (i.e., to induce the production of antibodies). A related polyarginine compound that we have developed, NP213, is not antigenic in rodents, and exposure does not lead to antibody production, unless the peptide is conjugated to a model antigen, such as keyhole limpet hemocyanin (KLH) (D. O’Neil, unpublished observation). Similarly, NP339 was well tolerated in mouse models of infection, further highlighting the lack of apparent toxicity—at least at the therapeutic doses tested and duration of therapy used in the present study. This highlights the potential of NP339 as a novel antifungal drug candidate.

In addition, similar to HDPs (46), NP339 did not elicit resistance *in vitro*. The peptide is rapidly fungicidal and targets the cell membrane, not a discrete intracellular target, and kills by a “physical” interaction with membrane components resulting in membrane lysis. In contrast, currently used antifungal drug classes are target molecule specific, and their use may promote resistance development, impeding therapeutic utility. For instance, two classes of the currently used antifungals target specific molecules within the fungal cell membrane: azoles (e.g., fluconazole) and polyenes (e.g., amphotericin B) (5, 47). The former inhibits lanosterol 14α-demethylase, a key enzyme of the ergosterol biosynthesis pathway encoded by *CYP51* in filamentous fungi and *ERG11* in *Candida* spp., while the latter bind to ergosterol, thus disrupting the cell membrane. Various resistance mechanisms to these drugs were reported and include mutations in the target-encoding gene (48, 49), altered expression of the target enzyme (50–52), or removal of the drug from the cell by efflux (53–55). Alternatively, antifungal drug use may allow the growth of inherently less susceptible strains (56). Indeed, we observed that while prolonged exposure to sub-MICs of some standard-of-care antifungals induced resistance in the tested fungi, no such resistance to NP339 developed. Of note, we did not observe NP339 cross-resistance in fungi that have acquired resistance to other classes of antifungals. That is reassuring for an antifungal drug candidate, as cross-resistance (e.g., resistance achieved by overexpression of efflux pumps or modification of cell structures [57, 58]), would limit its utility.

Drug-drug interactions should be considered when devising novel antifungals. Many of the currently used antifungal agents are processed in the liver, and their metabolism is affected by hepatic active drugs and *vice versa* (59). This is especially well documented for azoles: metabolic conversions of azoles are affected by coadministration of drugs that impact hepatic xenobiotic metabolism; conversely, azoles impair the metabolism of some coadministered drugs by collaterally inhibiting human cytochrome enzymes (60). For the former, rifampin (an antituberculosis drug) induces the activity of hepatic cytochrome P450 (CYP450) enzymes; this accelerates the metabolism of itraconazole and, consequently, decreases its antifungal activity (61). For the latter, inhibition of CYP450 34A by itraconazole inhibits the clearance of lovastatin (a cholesterol-lowering drug), thus increasing the risk of skeletal muscle toxicity (62). In contrast, exogenous peptides are not processed in the liver, which minimizes the risk of the involvement of NP339 in adverse drug-drug interactions (63, 64). This bodes well for its applicability in the most likely target patient population for NP339: i.e., chemotherapy and transplant recipients and otherwise immunocompromised and seriously ill individuals already subject to polypharmaceutical interventions.

Finally, the observations in rodent models of fungal infections further confirmed NP339 as an antifungal drug candidate. NP339 administration resulted in fungal burden reduction in the vaginal and oropharyngeal candidiasis models, as well as in the invasive pulmonary aspergillosis model. Of note, these effects were observed even though the NP339 dose, mode of administration, and fungal burden detection (by plating of homogenized tissue) had not been optimized for peptide drugs.

An important limitation of the current study is the inability to predict the actual NP339 efficacy and performance for clinical application based on *in vitro* characterization via traditional, often inappropriate, antimicrobial susceptibility tests performed in tissue culture medium. Specifically, these methods used for MIC determinations underestimate the activity of membrane-targeting antimicrobial peptides, with the apparent MIC values exceeding those of nonpeptide antimicrobials (37). Assays such as GI determination in blood are much more reflective of therapeutic potential. Furthermore, the tested microbes might react differently to peptide exposure within a specific microenvironment within the host compared with under *in vitro* conditions. This underscores the importance of peptide activity testing in biological matrices reflective of the infection site, such as the saliva, blood, etc. The above problems lead to under- or overestimation of the actual antimicrobial peptide activity (41, 65–67). Conversely, metabolic stability of therapeutic peptides and availability *in vivo* are difficult to predict (68–70). The strength of this study is the determination of the rapid, cidal antifungal mode of activity of a novel peptide therapy candidate and, for the first time, of peptide antifungal activity in human whole blood (and other biological matrices) by nonculture methods. The results of the current study strongly position NP339 as a novel antifungal peptide candidate with a broad therapeutic potential.

In conclusion, the broad spectrum of activity, high specificity for the fungal cell, and low apparent toxicity render NP339 an attractive drug candidate. We are currently actively pursuing its further development for application against specific fungal diseases.

## MATERIALS AND METHODS

### Reagents.

NP339 is a 2-kDa polyarginine obtained by solid-phase synthesis (Almac Sciences, Edinburgh, United Kingdom, and Polypeptide, Strasbourg, France) as a polyarginine acetate at >95% purity. The purity of the preparation was checked by high-performance liquid chromatography. Unless stated otherwise, NP339 was prepared as a 20-mg/ml stock in water. All other polyarginine peptides tested were synthesized by Almac Sciences or PepScan (Lelystad, Netherlands) and assayed similarly to NP339.

### Fungi and growth conditions.

All fungal strains tested are listed in Table S2 in the supplemental material. For general handling and unless stated otherwise, all strains were cultured on Sabouraud dextrose (SAB) agar slants (Sigma-Aldrich, Dorset, United Kingdom) for the indicated times prior to experiments: *Candida* strains were precultured at 35°C for 24 h, Aspergillus strains were precultured at 35°C for 48 h, Cryptococcus strains were precultured at 30°C for 24 h, *Exophiala* strains were precultured at 35°C for 72 h, *Scedosporium* strains were precultured at 35°C for 72 h, and *Mucorales* strains were precultured at 35°C for 48 h.

### Electron microscopy.

For the TEM analysis, an overnight culture of C. albicans AM2003-0182 was diluted to a density of 1 × 10^8^ cells/ml in 2× RPMI 1640 medium, and the mixture was incubated at 35°C for 1 h. The cells were then washed three times in sterile water and once in 0.01 M sodium phosphate buffer. Next, 1 ml of 1× MIC of NP339 (final concentration, 4 mg/liter), caspofungin (final concentration, 0.5 mg/liter), or human β-defensin 2 (final concentration, 25 mg/liter) was added, and the incubation continued at 30°C for 30 min. The cells were then fixed in 2.5% (vol/vol) glutaraldehyde and stored at 4°C before analysis. For TEM analysis, the cells were dehydrated in an ethanol and acetone series before being embedded in wax resin, stained with uranyl acetate/lead citrate stains to improve contrast, sectioned to a 90-nm thickness, and mounted onto copper grids for TEM analysis. TEM micrographs were acquired using a JEM-1400 Plus JEOL transmission electron microscope. Microscopy was performed in the Microscopy and Histology Core Facility at the University of Aberdeen (United Kingdom).

For the SEM analysis, 1 ml of a suspension of 2 × 10^4^ Aspergillus conidia was incubated in RPMI 1640 medium (Sigma) in a 24-well polystyrene plate containing a 0.2-μm-pore Whatman polycarbonate filter for 48 h at 35°C. The medium was then removed, 1 ml of RPMI 1640 medium containing 250 mg/liter of lipid-tagged NP339 was added, and the samples were incubated for 1 h at 30°C. The medium was removed, 1 ml of 2% (vol/vol) glutaraldehyde in 0.1 M sodium phosphate buffer was added, and the samples were incubated for at least 12 h at 4°C to fix the cells. The filters were then removed, rinsed three times with 0.1 M sodium phosphate buffer for 5 min each, and placed in 1% (wt/vol) osmium tetroxide in distilled H_2_O for 1 h. They were next washed three times with water for 5 min each. The samples were dehydrated in a graded ethanol series (70, 80, and 90%, [vol/vol]) for 10 min in each dilution and then three times in 100% ethanol for 10 min each. The filters were placed in hexamethyldisiloxane (Sigma) for 10 min and then left to dry overnight. All samples were mounted on aluminum stubs and coated with gold. Images were acquired using a Zeiss EVO MA10 scanning electron microscope (Zeiss, Cambridge, United Kingdom) at the Electron Microscopy Services Department at the University of Aberdeen.

### Charge neutralization experiments.

The culture of C. albicans SC5314 was grown at 30°C overnight and prepared to a 0.5 McFarland standard, as described for the time-kill experiments. For the experiment, 16% (wt/vol) PASA solution was prepared in sterile distilled H_2_O (sdH_2_O) and serially diluted to final concentrations of 2, 4, 8, and 16%. Then, 250 μl of NP339 solution in sterile distilled H_2_O (16 or 32 μg/ml) was added to 250 μl of PASA dilution, and the samples were incubated for 30 min at 37°C. Next, 500 μl of the prepared cells was added, and the incubation continued at 37°C. Samples were removed at 0, 0.5, 1, and 2 h, diluted in sterile water, plated on SAB agar, and incubated at 30°C for 24 h, and the resultant colonies were counted.

### Peptide specificity and penetration assays.

The cell-penetrating peptide Arg9 was purchased from Bachem AG (Bubendorf, Switzerland). The C termini of NP339 and Arg9 peptides were labeled with BODIPY FL EDA in a reaction mixture containing 2 mM peptide, 10 mM BODIPY FL EDA, 10 mM 1-ethyl-3-(3-dimethylaminopropyl)carbodiimide, and 5 mM *N*-hydroxysulfosuccinimide (all from Fisher Scientific UK, Ltd., Loughborough, United Kingdom) in 0.1 M 2-(*N*-morpholino)ethanesulfonic acid buffer (MOPS; pH 5.0) (Melford Laboratories, Ltd., Ipswich, United Kingdom) by stirring gently for 3 h at 25°C. Unbound label and excess reagents were removed using SpinOUT GT-100 columns (Web Scientific, Ltd., Crewe, United Kingdom), and the amount of peptide was quantified using a Micro BCA (bicinchoninic acid) protein assay kit (Fisher Scientific UK, Ltd.).

C. albicans SC5314 and A. fumigatus AM2002/0066 were grown for 24 or 48 h, respectively, on SAB agar (Sigma) slants at 35°C. A. fumigatus AM2002/0066 cells were harvested from the slant into 0.15 M NaCl (Fisher Scientific UK, Ltd.), and the conidia were isolated by passing through an EASYStrainer cell sieve (70-μm pore; Greiner Bio-One, Gloucester, United Kingdom). They were then allowed to germinate by inoculating 1 × 10^5^ CFU/ml conidia into RPMI 1640 medium and incubating them at 25°C for 18 to 20 h. C. albicans SC5314 cells were harvested from the slant and then allowed to form hyphae by inoculating 1 × 10^5^ CFU/ml cells into RPMI 1640 medium and incubating them at 37°C for 4 h. PBMCs were isolated from fresh human whole blood using Leucosep (Greiner Bio-One) tubes according to the manufacturer’s instructions and diluted to a concentration of 1 × 10^5^ cells/ml in RPMI 1640. BODIPY-labeled peptides (20 μM) were added to 100 μl of cells (PBMCs or fungi) in 16-well chamber slides (Thistle Scientific, Ltd., Glasgow, United Kingdom) and incubated at 37°C for 60 min. The samples were then analyzed by fluorescence and light microscopy using an Axiovert 40 CXL microscope (Carl Zeiss, Ltd.) with a 40× objective and filter set 10 (λ_Ex_ = 450 to 490 nm; λ_Em_ = 515 to 565 nm) for fluorescence microscopy. Cell images were captured after an 800-ms exposure.

### MIC determinations.

MIC values were determined following the CLSI-approved *Reference Method for Broth Dilution Antifungal Susceptibility Testing of Yeasts* (approved standard M27-A3) (32) in RPMI 1640 medium (Sigma, Dorset, United Kingdom) at 35°C. For all filamentous fungi, the CLSI standard M38-A2 (33) was followed, in RPMI 1640 medium at 35°C. Cell growth of all fungi was followed by measuring the optical density at 530 nm (OD_530_) using the Powerwave XS plate reader (Biotek, Swindon, United Kingdom). The MIC values are reported for the 24-h time point for *Candida* spp., Aspergillus spp., and *Mucorales* spp., for the 48-h time point for Cryptococcus spp., for the 48-h time point for *Scedosporium* spp., and for the 72-h time point for *Exophiala* spp.

### Time-kill assays in culture media.

For *Candida* spp., the cells were grown for at least 18 to 24 h at 30°C, until visible growth was observed. The cells were then centrifuged and resuspended in sterile distilled H_2_O and diluted to the 0.5 McFarland standard density using 2× RPMI 1640 medium. The tested antifungals were prepared at twice the required concentration. Then, 1 ml of the inoculum was added to 1 ml of the antifungal solution and incubated at 30°C. Every hour over the course of 8 h, 20 μl of the culture was removed and 10-fold serial dilutions were prepared (from 1 × 10^−1^ to 1 × 10^−7^). Then, 50 μl of each dilution was plated on SAB agar (Sigma) in triplicate. The plates were incubated at 30°C until colonies were observed on the control plates with vehicle-treated fungi. The colonies were counted, and the percentage killed was determined based on the reduction in the number of CFU/ml in the treated samples compared with the vehicle-treated, untreated controls.

For A. fumigatus, a subculture was grown on a SAB agar (Sigma) slant for 72 h at 35°C until sporulation. A. fumigatus cells were harvested from the slant into 0.15 M NaCl (Fisher Scientific UK, Ltd.), and conidia were isolated by passing through an EASYStrainer cell sieve (70-μm pore; Greiner Bio-One, Gloucester, United Kingdom) The conidial suspension was diluted to 1 × 10^5^ CFU/ml in 2× antibiotic medium 3 (Oxoid). For germling analysis, the conidial suspension was incubated for 8 h at 35°C prior to analysis; for conidial experiments, it was used immediately. The experiment was performed as described for *Candida* spp.

### Time-kill assay in saliva.

A subculture of C. albicans MD (oral isolate) was grown overnight at 30°C on SAB medium. The cells were then resuspended in sterile deionized H_2_O and diluted to a density that was 20× higher than the 0.5 McFarland standard. NP339 was prepared at 20× the required concentration in sterile distilled H_2_O. Human saliva was collected in the morning, prior to eating or brushing teeth, and filter sterilized before use by passing through a 70-μm-pore EasyStrainer (Greiner Bio-One), followed by passing through a 40-μm-pore EasyStrainer (Greiner Bio-One). The saliva was then sequentially syringe filtered through a 0.45-μm-pore filter and a 0.22-μm-pore filter. For each treatment, 50 μl of 20× inoculum and 50 μl of 20× NP339 were added to 900 μl of filter-sterilized saliva, and the samples were incubated at 30°C. At the specified time points, 10 μl of the mixture was removed, and 10-fold serial dilutions were prepared in sterilized distilled H_2_O, for a range of dilutions between 1 × 10^−2^ and 1 × 10^−7^. Then, 50 μl of each dilution was plated on SAB agar in triplicate. The plates were incubated at 30°C until colonies were observed on control plates (untreated saliva-exposed cells). Colonies were counted, and the percentage killed determined based on the reduction in CFU/ml in the treated samples relative to the untreated controls.

### BioFlux flow system experiments.

C. albicans MD was grown at 30°C on SAB agar. The cells were removed by taking a loopful and resuspending it in sterile water. The cells were then washed three times with sterile water and resuspended in RPMI 1640 tissue culture medium at a density of 1 × 10^7^ cells/ml. The BioFlux 200 system (Fluxion, South San Francisco, CA, USA) was primed with sterile RPMI 1640 prior to the inoculation of cells. The cells were placed in the out-well of a 24-well BioFlux 200 plate, and a pressure of 1 dyne was applied across the sealed plate from the out-well to the in-well for 10 s, until the cells could be seen within the microfluidic chamber. The pressure was then removed, and the cells were allowed to adhere for 1.5 h on a heated stage at 37°C. Human saliva from volunteers was centrifuged at 1,400 rpm for 5 min to remove any debris and then passed through a 70-μm-pore EasyStrainer (Greiner Bio-One), followed by passing through a 40-μm-pore EasyStrainer (Greiner Bio-One). The saliva was then sequentially syringe filtered through a 0.45-μm-pore filter and a 0.22-μm-pore filter. The filter-sterilized saliva was incubated at 30°C until use. NP339 (125 mg/liter) was immobilized in 90% saliva. Once the cells had adhered in the microfluidic chamber for 1.5 h, NP339 in 90% saliva was added to the in-well of the BioFlux plate, placed on the heated stage at 37°C, and a pressure of 0.5 dyne was applied from the in-well to the out-well for 20 h. The microfluidic channels were photographed every 5 min to determine biofilm formation.

### GI determination.

Human whole blood was obtained from healthy volunteers. A. fumigatus AM2002/0066 conidia were harvested from mature cultures in phosphate-buffered saline (PBS). For the experiment, citrated human whole blood was first mixed at a ratio of 9:1 with a suspension of A. fumigatus AM2002/0066 conidia and then treated with different concentrations of NP339 prepared in PBS. This resulted in a final concentration of 1 × 10^5^ conidia/ml in 85% (vol/vol) blood. The mixtures (850 μl of blood, 100 μl of inoculum, and 50 μl of NP339) were incubated at 37°C for 24 h, resuspended by gentle pipetting, and centrifuged at 1,500 × *g* for 5 min. The supernatant (plasma) was retrieved and diluted 1:100 in sdH_2_O before processing as per the Platelia galactomannan enzyme-linked immunosorbent assay (ELISA) protocol (Invitrogen, Fisher Scientific UK, Ltd.). The GI was calculated using kit standards, and GI data were normalized to those of the PBS sham-treated control (71).

### Resistance development experiments.

Antifungals were prepared at 0.5× MIC in sterile distilled H_2_O and stored at −20°C for the duration of the assay. The fungal inoculum was prepared according to the CLSI standard M27-A3 (32). For the analysis, 100 μl of the antifungal stock was added to a 96-well polystyrene plate in triplicate. Then, 100 μl of the inoculum was added, for the final antimicrobial concentration of 0.25× MIC. Every 24 h, the sample OD_530_ was measured using Powerwave XS plate reader (Biotek). If ΔOD_530_, calculated as the difference between treated and untreated (medium-only) samples, exceeded 0.1, the culture was passaged by dilution to the CLSI standard inoculum in 2× RPMI 1640 medium and mixed with 100 μl of the 0.5× MIC solution of the antifungal. The passaging was repeated 30 times. Every five passages, a full MIC analysis was done, as specified above. After 30 passages, a full NP339 MIC analysis was done for all strains, including those that had developed resistance to antifungal agents other than NP339.

### NP339 cytotoxicity.

PBMCs were isolated from fresh human whole blood using Leucosep tubes (Greiner Bio-One), according to the manufacturer’s instructions. The cells were suspended in RPMI 1640 medium (5 × 10^6^ cells/ml) and transferred to 96-well plate (5 × 10^5^ cells/well). NP339 solutions were prepared in prewarmed RPMI 1640 medium and added to the cell suspension. The plates were incubated at 37°C under 5% CO_2_ for 2 h. Toxicity was determined using the Molecular Probes LIVE/DEAD Viability/Cytotoxicity kit (Fisher Scientific UK, Ltd.). As the kill control, 0.1% (vol/vol) Triton X-100 was used. Untreated cells were the live control.

Lung epithelial cells (A549 cells; ATCC [distributed by LGC Promochem, Teddington, United Kingdom]) were cultured in a 96-well plate in Ham’s F-12K medium (Fisher Scientific UK, Ltd.) supplemented with 10% (vol/vol) fetal bovine serum. The cells were incubated under 5% CO_2_ at 37°C until 90% confluent. NP339 solutions and 0.25% SDS (the kill control) were prepared in prewarmed RPMI 1640 medium. Once the cells reached 90% confluence, the culture medium was removed from the 96-well plate, taking care not to disturb the cells, and 100 μl of NP339 solution was added to the cell monolayer. The plates were incubated at 37°C under 5% CO_2_ for 3 h. Toxicity was determined using the Molecular Probes LIVE/DEAD Viability/Cytotoxicity kit, as per the manufacturer’s instructions. As the kill control, 0.25% (wt/vol) SDS was used. Untreated cells were the live control (cells incubated with the medium).

### Hemolysis assay.

The hemolysis assay was performed in 96-well V-bottom microplates. Stock solutions of NP339 were prepared at twice the required maximum concentration in Hanks’ balanced salt solution (HBSS; Fisher Scientific UK, Ltd.). A positive control of sterile H_2_O and a negative control of HBSS were also prepared. Human whole blood was obtained from healthy volunteers by a trained phlebotomist. The blood samples were centrifuged at 800 × *g* for 15 min, and the plasma and PBMC layer were removed. Red blood cells were then washed with Dulbecco’s PBS (DPBS; Fisher Scientific UK, Ltd.) and centrifuged at 400 × *g* for 15 min. The DPBS layer was removed, and the process was repeated until the supernatant became clear after the centrifugation. For the experiment, 200 μl of a solution of a double the desired concentration of NP339 was added to the first row of a V-bottom 96-well plate and serially diluted down the plate with 100 μl of HBSS, resulting in 100 μl of double-strength test solution in each well. Next, 1 ml of red blood cell suspension was added to 10 ml of DPBS; 100 μl of the diluted red blood cell preparation was then added to each well. The plate was incubated for 3 h at 37°C under 5% CO_2_. Following the incubation, the supernatant was transferred to a fresh flat-bottom 96-well plate, and the sample OD_578_ was read using a Biotek Powerwave XS plate reader (Biotek). The hemolysis percentage was calculated as (expOD − negOD)/(posOD − negOD), where expOD is the mean experimental OD, negOD is the mean negative-control OD, and posOD is the mean positive-control OD.

### *In vivo* models of infection.

In all experiments, the animals were housed 4 or 5/cage in sterilized individual ventilated cages exposing the mice at all times to HEPA-filtered sterile air. Food and water were available *ad libitum*, and sterile aspen chip bedding was changed at least once weekly. The room temperature was 22 ± 1°C, with a relative humidity of 60% and maximum background noise of 56 dB. Mice were exposed to 12-h light/dark cycles. Before the experiments, the animals were randomly divided into groups. After the experiments, the animals were sacrificed by humane euthanasia by delivering 0.5 ml of 200 mg/ml pentobarbital solution via intraperitoneal injection. For quantification of fungal burden, samples were diluted appropriately and then quantitatively cultured on to SAB containing 0.05 mg/ml chloramphenicol and incubated at 37°C for 24 to 48 h before enumeration.

### (i) Murine model of systemic candidiasis.

Male CD1 mice (*n* = 24 mice, 6 to 8 weeks of age, average weight of 23.5 g) were obtained from Charles River, Margate, United Kingdom. All mice were infected with C. albicans FA6862 (approximately 1.1 × 10^4^ CFU/mouse in 0.2 ml) by intravenous injection into the lateral tail vein. The mice were randomly divided into three groups (*n* = 8/group): the control group (10 ml/kg of body weight 0.9% saline administered intravenously twice daily), the NP339 group (NP339 in 0.9% saline administered intravenously in 10 ml/kg at 3 mg/kg twice daily), and the caspofungin group (drug in 0.9% saline administered intraperitoneally in 10 ml/kg at 1 mg/kg once daily). Caspofungin was from Merck Sharp & Dohme, Ltd., Hertfordshire, United Kingdom. The mice were culled 29 h postinfection, and fungal kidney burdens were determined by plating as previously described.

### (ii) Murine model of vaginal candidiasis.

Female BALB/c mice (*n* = 24 mice, 6 to 8 weeks of age, weight of 16 to 18 g) were obtained from Charles River, Margate, United Kingdom. All mice were topically infected with C. Albicans 529L (4.5 × 10^6^ CFU/mouse in 0.025 ml) by intravaginal injection under temporary inhaled anesthesia (2.5% isoflurane for 3 to 4 min). The mice were randomly divided into three groups (*n* = 8/group): the control group (0.05 ml of 65% [vol/vol] polyethylene glycol [PEG] 14000 administered vaginally once daily), the NP339 group (0.05 ml NP339 in 65% [vol/vol] PEG 14000 administered in sterile distilled water at 1 mg/kg vaginally once daily), and the clotrimazole group (0.05 ml, as purchased at 1% [wt/vol], administered vaginally once daily). Clotrimazole (Canetsen) was from Bayer, Leverkusen, Germany. At 3 days postinfection, fungal burdens in vaginal lavage samples were determined as described above.

### (iii) Murine model of oropharyngeal candidiasis.

Male CD1 mice (*n* = 24 mice, 6 to 8 weeks of age, average weight of 24.6 g) were obtained from Charles River, Margate, United Kingdom. All mice were infected with C. albicans FA6862 (approximately 4.0 × 10^8^ CFU/mouse topically in the oral cavity via 20 min of exposure to a swab soaked in the fungal suspension), under temporary inhaled anesthesia (1.5 to 2% isoflurane). The mice were randomly divided into three groups (*n* = 8/group): the control group (∼0.1 ml of PEG 300-soaked swab administered topically [oral application with a 10-min exposure administered with a swab under anesthesia] once daily); the NP339 group (NP339 in PEG 300 administered topically at 1 mg/kg once daily), and the fluconazole group (drug administered orally at 5 mg/kg once daily). Fluconazole was from Pfizer UK, Tadworth, United Kingdom. At 4 days postinfection, fungal burdens in swab fluid were determined by plating as described before.

### (iv) Murine model of invasive pulmonary aspergillosis.

Male CD1 mice (*n* = 24 mice, 8 to 10 weeks of age, average weight of 27.0 g) were obtained from Charles River, Margate, United Kingdom. Before the experiment, the mice were immunosuppressed, as follows: days −4 to +1, subcutaneously injected with ceftriaxone (50 mg/kg), prepared as a 5-mg/ml solution in sterile saline for injection; days –4 and –1, intraperitoneally injected with cyclophosphamide (150 mg/kg), prepared as a 20-mg/ml solution dosed at 7.5 ml/kg in sterile saline for injection; and day –1, subcutaneously injected with 175 mg/kg cortisone acetate, prepared as a 17.5-mg/ml solution in sterile PBS–0.05% Tween 80. On day 0, all mice were infected with A. fumigatus A1163 (7.2 × 10^4^ CFU/mouse in 0.04 ml) by intranasal administration under anesthesia (2.5% isoflurane for 3 to 4 min). The mice were randomly divided into three groups (*n* = 8/group): control (nebulized WFI administered twice daily for 10 min), the NP339 group (nebulized NP339 in WFI administered at 5 mg/kg, twice daily), and the AmBisome group (AmBisome in 5% glucose infusion solution [Baxter] administered intravenously in 10 ml/kg at 5 mg/kg once daily). AmBisome was from Gilead Sciences, Inc., San Dimas, CA. Fungal lung burdens were determined at the clinical endpoint as previously described.

### Ethical statement.

Written informed consent was obtained for the provision of whole blood from all volunteer donors. All animal experiments were performed by a third party contractor under UK Home Office License.

### Statistical analysis.

Unless otherwise stated, all experiments were prepared at least in triplicate with three technical replicates. All statistical analyses were performed using GraphPad Prism v7 or higher (GraphPad Software, San Diego, CA). Statistical significance of *in vivo* data was assessed by Mann-Whitney analyses relative to vehicle-treated control. One-way analysis of (ANOVA) with Dunnett’s multiple-comparison test was used to analyze GI data. A *P* value of ≤0.05 was considered to indicate statistical significance.

### Data availability.

All data are presented in the manuscript and the associated supplemental material files.
