# Supplementary material for: Preliminary Characterization of NP339, a Novel Polyarginine Peptide with Broad Antifungal Activity
Source: Antimicrob Agents Chemother. 2021 Jul 16;65(8):e02345-20. doi: 10.1128/AAC.02345-20 (PMC8284473; doi:10.1128/AAC.02345-20)
Supplement: Supplemental file 1 — Supplemental Tables S1 and S2. Download AAC02345-20_Supp_1_seq12.pdf, PDF file, 0.6 MB [file aac02345-20_supp_1_seq12.pdf]

SUPPLEMENTAL MATERIAL FOR PUBLICATION

Preliminary Characterisation of NP339, a Novel Polyarginine Peptide with Broad Antifungal Activity

[1]Vanessa Duncan, [1]Daniel Smith, [1]Laura Simpson, [1]Emma Lovie, [1]Laura Katvars, [1]Leon Berge, [1]Jennifer Robertson, [1,2]Shane Smith, [2]Carol Munro, [1]Derry Mercer and [1]Deborah O'Neil#

[1]NovaBiotics Ltd., Silverburn Crescent, Bridge of Don, Aberdeen, AB23 8EW, UK

[2]The University of Aberdeen, Institute of Medical sciences, Foresterhill Health Campus Foresterhill Road, Aberdeen, AB25 2ZD, UK

Running Head: Novel Antifungal Peptide NP339

#Address correspondence to Deborah O'Neil (Deborah@novabiotics.co.uk)

SUPPLEMENTAL TABLES

TABLE S1 NP339 has a broad antifungal activity

Fungal species	Number of strains tested	MIC range (mg/l)	MIC_{50} (mg/l)	MIC_{90} (mg/l)
Candida albicans	24	1–64	1	4
Candida glabrata	20	2–1024	32	32
Candida parapsilosis	23	1–524	32	32
Candida krusei	21	2–64	4	4
Candida tropicalis	17	1–64	1	2
Candida auris	6	1–8	n.d.	n.d.
Candida dubliniensis	8	2–4	n.d.	n.d.
Cryptococcus neoformans	14	1–4	2	2
Cryptococcus gattii	2	1–2	n.d.	n.d.
Scedosporium apiospermum	6	<0.128–256	n.d.	n.d.
Aspergillus fumigatus	25	16–1024	16	32
Aspergillus terreus	16	<8–128	n.d.	n.d.
Aspergillus flavus	10	>256	n.d.	n.d.
Exophiala dermatitidis	6	2–32	n.d.	n.d.
Rhizopus oryzae	1	8	n.d.	n.d.
Rhizopus microspores	1	36	n.d.	n.d.
Rhizomucor pusillus	1	2	n.d.	n.d.
Mucor circinelloides	1	8	n.d.	n.d.
Cunninghamella bertholletiae	1	64	n.d.	n.d.
Absidia corymbifera	1	3	n.d.	n.d.

MIC (mg/l) of NP339 against a variety of fungal pathogens. MIC range indicates the concentration required to kill the members of the group, whilst MIC_{50} and MIC_{90} ranges

indicate the concentrations required to inhibit 50% and 90% of the strains, respectively. n.d., (not determined) indicates strains whereby less than nine isolates were tested, therefore the MIC_{50} and MIC_{90} could not be determined.

TABLE S2 Fungal strains used in the current study (file in .xlsx format)

Genus	Species	Strain
Candida	***albicans***	ATCC24433
Candida	***albicans***	SC5314
Candida	***albicans***	ATCC90028
Candida	***albicans***	NCTC3179
Candida	***albicans***	73/034
Candida	***albicans***	AM2003-020
Candida	***albicans***	AM2003-0191
Candida	***albicans***	AM2003-0069
Candida	***albicans***	AM2003-0100
Candida	***albicans***	AM2003-0182
Candida	***albicans***	AM2004-0025
Candida	***albicans***	AM2005-0377
Candida	***albicans***	HUN68
Candida	***albicans***	IHEM3742
Candida	***albicans***	IHEM16614
Candida	***albicans***	IHEM16945
Candida	***albicans***	IHEM16972
Candida	***albicans***	L1086
Candida	***albicans***	RV4688
Candida	***albicans***	S20122.073
Candida	***albicans***	S20152.013
Candida	***albicans***	S20152.016
Candida	***albicans***	S20176.079
Candida	***albicans***	T101
Candida	*auris*	DSMZ21092
Candida	*auris*	CBS12767
Candida	*auris*	CBS10313
Candida	*auris*	CBS12372
Candida	*auris*	CBS12373
Candida	*auris*	CBS12766

Genus	Species	Strain
Candida	*glabrata*	AM2002-0085
Candida	*glabrata*	AM2002-0088
Candida	*glabrata*	AM2007-0113
Candida	*glabrata*	AM2007-0114
Candida	*glabrata*	AM2007-0015
Candida	*glabrata*	AM2007-0116
Candida	*glabrata*	AM2007-0117
Candida	*glabrata*	AM2007-0118
Candida	*glabrata*	AM2009-0119
Candida	*glabrata*	AM2007-0120
Candida	*glabrata*	AM2007-0121
Candida	*glabrata*	AM2007-0122
Candida	*glabrata*	AM2007-0125
Candida	*glabrata*	AM2007-0126
Candida	*glabrata*	AM2007-0127
Candida	*glabrata*	AM2007-0128
Candida	*glabrata*	AM2007-0129
Candida	*glabrata*	AM2007-0130
Candida	*glabrata*	NCPF3943
Candida	*glabrata*	NCPF3831
Candida	*krusei*	ATCC6258
Candida	*krusei*	NCPF3953
Candida	*krusei*	AM30308.03.05
Candida	*krusei*	AM2005-0492
Candida	*krusei*	AM2005-0494
Candida	*krusei*	AM2005-0496
Candida	*krusei*	AM2007-0106
Candida	*krusei*	AM2007-0107
Candida	*krusei*	AM2005-0498
Candida	*krusei*	AM2005-0525
Candida	*krusei*	AM2005-0531
Candida	*krusei*	AM2006-0127
Candida	*krusei*	AM31194.04.05
Candida	*krusei*	AM30332.04.05
Candida	*krusei*	AM30455.04.05
Candida	*krusei*	AM30274.03.05
Candida	*krusei*	AM2007-0105
Candida	*krusei*	AM2007-0102
Candida	*krusei*	AM2007-0103
Candida	*krusei*	AM2007-0107
Candida	*krusei*	AM2007-0109

Genus	Species	Strain
Candida	*parapsilosis*	ATCC90018
Candida	*parapsilosis*	ATCC22019
Candida	*parapsilosis*	AM2005-0238
Candida	*parapsilosis*	AM2005-0225
Candida	*parapsilosis*	AM2007-0137
Candida	*parapsilosis*	AM2005-0111
Candida	*parapsilosis*	AM2005-0358
Candida	*parapsilosis*	AM2007-0135
Candida	*parapsilosis*	AM2005-0099
Candida	*parapsilosis*	AM2005-0304
Candida	*parapsilosis*	AM2007-0132
Candida	*parapsilosis*	AM2007-0138
Candida	*parapsilosis*	AM205-0112
Candida	*parapsilosis*	AM2007-0139
Candida	*parapsilosis*	AM2007-0133
Candida	*parapsilosis*	AM2005-0337
Candida	*parapsilosis*	AM2005-0239
Candida	*parapsilosis*	AM2005-0233
Candida	*parapsilosis*	AM2007-0136
Candida	*parapsilosis*	AM2007-0134
Candida	*parapsilosis*	AM2007-0131
Candida	*parapsilosis*	AM2005-0242
Candida	*parapsilosis*	AM2005-0237
Candida	*tropicalis*	ATCC750
Candida	*tropicalis*	AM2007-0111
Candida	*tropicalis*	AM2005-0087
Candida	*tropicalis*	AM2007-0110
Candida	*tropicalis*	AM2004-0089
Candida	*tropicalis*	AM2007-0112
Candida	*tropicalis*	AM2004-0088
Candida	*tropicalis*	AM2004-0090
Candida	*tropicalis*	AM2004-0091
Candida	*tropicalis*	AM2004-0094
Candida	*tropicalis*	AM2005-0560
Candida	*tropicalis*	AM2005-0563
Candida	*tropicalis*	AM2005-0564
Candida	*tropicalis*	AM2005-0565
Candida	*tropicalis*	AM2005-0551
Candida	*tropicalis*	AM2005-0553
Candida	*tropicalis*	AM2005-0558

Genus	Species	Strain
Candida	*dublineisis*	81/060
Candida	*dublineisis*	90/033
Candida	*dublineisis*	AM2005-0178
Candida	*dublineisis*	J931021
Candida	*dublineisis*	AM2002-0024
Candida	*dublineisis*	AM2005-024
Candida	*dublineisis*	AM2005-0166
Candida	*dublineisis*	AM2007-0178
Cryptococcus	*neoformans*	NCPF8224
Cryptococcus	*neoformans*	JEC21
Cryptococcus	*neoformans*	ATCC32609
Cryptococcus	*neoformans*	ATCC-MYA-4093
Cryptococcus	*neoformans*	DSMZ11959
Cryptococcus	*neoformans*	NCPF8131
Cryptococcus	*gattii*	R265
Cryptococcus	*gattii*	CBS6993

Genus	Species	Strain
Aspergillus	*fumigatus*	AM2002-0066
Aspergillus	*fumigatus*	ATCC-MYA-3626
Aspergillus	*fumigatus*	ATCC-MYA-3627
Aspergillus	*fumigatus*	A1163
Aspergillus	*fumigatus*	NCPF7367 (AF293)
Aspergillus	*fumigatus*	AF23
Aspergillus	*fumigatus*	NCPF2939
Aspergillus	*fumigatus*	AFG AF20
Aspergillus	*fumigatus*	AFG AF21
Aspergillus	*fumigatus*	AFG AF22
Aspergillus	*fumigatus*	AFG AF23
Aspergillus	*fumigatus*	AFG AF60
Aspergillus	*fumigatus*	AFG AF69
Aspergillus	*fumigatus*	AFG AF70
Aspergillus	*fumigatus*	AFG AF78
Aspergillus	*fumigatus*	AFG2002-0062
Aspergillus	*fumigatus*	AFG2002-0063
Aspergillus	*fumigatus*	AFG2002-0065
Aspergillus	*fumigatus*	AFG2002-067
Aspergillus	*fumigatus*	AFG2004-0040
Aspergillus	*fumigatus*	AFG2004-0042
Aspergillus	*fumigatus*	AFG2004-0043
Aspergillus	*fumigatus*	AFG2004-0045
Aspergillus	*fumigatus*	AFG2004-0066
Aspergillus	*fumigatus*	DM2006 1255a

Genus	Species	Strain
Aspergillus	*terreus*	AFG01-280
Aspergillus	*terreus*	AFG01-601
Aspergillus	*terreus*	AFG01-644
Aspergillus	*terreus*	AFG01-720
Aspergillus	*terreus*	AFG01-890
Aspergillus	*terreus*	AFG01-1322
Aspergillus	*terreus*	AFG01-1459
Aspergillus	*terreus*	AFG01-1908
Aspergillus	*terreus*	AFG01-2034
Aspergillus	*terreus*	AFG01-2376
Aspergillus	*terreus*	AFG01-2441
Aspergillus	*terreus*	AFG01-2856
Aspergillus	*terreus*	AFG01-3223
Aspergillus	*terreus*	AFG01-1714
Aspergillus	*terreus*	AFG01-R3260
Aspergillus	*terreus*	ATCC-MYA-3633
Aspergillus	*terreus*	NCPF2729

Aspergillus	*flavus*	AFG 01-1100
Aspergillus	*flavus*	AFG 01-1170
Aspergillus	*flavus*	AFG 01-1285
Aspergillus	*flavus*	AFG 01-1309
Aspergillus	*flavus*	AFG 01-1554
Aspergillus	*flavus*	AFG 01-2455
Aspergillus	*flavus*	AFG R-3283
Aspergillus	*flavus*	ATCC-MYA-3631
Aspergillus	*flavus*	ATCC204304
Aspergillus	*flavus*	NCPF7117
Aspergillus	*niger*	01-1494

Genus	Species	Strain
Scedosporium	*apiospermum*	ATCC-MYA-3634
Scedosporium	*apiospermum*	ATCC-MYA-3635
Scedosporium	*apiospermum*	NCPF2869
Exophiala	*dermatitidis*	CA01
Exophiala	*dermatitidis*	SP4002
Exophiala	*dermatitidis*	SP4493
Exophiala	*dermatitidis*	SP5884
Exophiala	*dermatitidis*	SP5973
Exophiala	*dermatitidis*	SP6339
Mucor	*circinelloides*	NCPF2708
Rhizopus	*microsporus*	NCPF2776
Rhizopus	*oryzae*	NCPF2504
Rhizopus	*pusillus*	NCPF2265
Cunninghamella	*bertholletiae*	NCPF2878
Absidia	corymbifera	NCPF2326
